# Supplementary material for: Causal interventions in bond multi-dealer-to-client platforms
Source: PLoS One. 2026 Jan 27;21(1):e0341369. doi: 10.1371/journal.pone.0341369 (PMC12844515; doi:10.1371/journal.pone.0341369)
Supplement: S1 Appendix — Complete description of cleaning, filters, and outlier removal. (PDF) [file pone.0341369.s001.pdf]

S1 File. Data preprocessing

We apply the following preprocessing steps before model training and evaluation:

1. **Anonymization:** client identifiers are replaced with non-reversible pseudonyms; no personally identifiable information is included in the working dataset.
2. **Price reconstruction for yield-quoted bonds:** when the RfQ is quoted in yield, we compute an approximate clean price using the quoted yield and standard conventions.
3. **Mandatory fields:** we drop RfQs with missing price or missing days-to-settlement.
4. **Spot normalization:** for RfQs with days-to-settlement different from two, we convert to spot price (T+2 convention) for consistency across observations.
5. **Universe filter:** we restrict the sample to Italian government bonds and exclude Treasury bills.
6. **Train/validation/test split:** we split the dataset into 75% / 15% / 15%, reserving the test set for final performance reporting and using the validation set for model selection and hyperparameter tuning.
7. **Outlier removal:** we remove outliers from the train set beyond 0.6 interquartile range (IQR) in the relevant pricing variables (e.g., spread), to mitigate undue influence of extreme observations.
8. **Dealer-count filter:** we retain only RfQs with 5, 10, 15, 20, or 24 invited dealers. These equally spaced values correspond to modes in the empirical distribution; focusing on them yields a large, homogeneous subset and reduces the number of parameterizations for the generative model from 25 to 5 (one per dealer count).
