# Supplementary material for: Causal interventions in bond multi-dealer-to-client platforms
Source: PLoS One. 2026 Jan 27;21(1):e0341369. doi: 10.1371/journal.pone.0341369 (PMC12844515; doi:10.1371/journal.pone.0341369)
Supplement: S1 Table — Summary statistics of the RFQ dataset (size, period, sides, dealers, notional, frequency). (PDF) [file pone.0341369.s002.pdf]

S1 Table. Data summary

After applying the preprocessing steps, this is the dataset that we use for the study:

**Characteristic**	**Value**
Number of RfQs	102,437
Period covered	Jun 2020 - Jun 2022
Number of unique clients	585
Number of unique bonds (ISINs)	113
Bond types	Italian Government Bonds
RfQ notional (mean $\pm$ std)	EUR 1.38 $\pm$ 4.62 million
Distribution of RfQ sides	47% Buy, 53% Sell
Average number of dealers per RfQ	10
Average trading frequency per ISIN	9 RfQs / week
Volume coverage	EUR 142 billion total requested

Table 1: Summary of the proprietary dataset of RfQs used in the study
