# Supplementary material for: Causal interventions in bond multi-dealer-to-client platforms
Source: PLoS One. 2026 Jan 27;21(1):e0341369. doi: 10.1371/journal.pone.0341369 (PMC12844515; doi:10.1371/journal.pone.0341369)
Supplement: S2 Table — Reference table. (PDF) [file pone.0341369.s003.pdf]

S2 Table. Notation

α	Client's price sensitivity
ACE	Average Causal Effect: Difference in outcomes between treated and untreated units
Axe	Pre-existing position the dealer wishes to buy or sell, so it is discounted
BBSS	Balanced Brier Skill Score: Metric for evaluating probabilistic calibration
BF	Bond Features: Attributes like coupon, DV01, maturity, etc.
CF	Client Features: Characteristics like client type, geography
δ	Spread quoted by the reference dealer with respect to a market mid-price
δ_{dealer}	Spread quoted by a dealer in competition
δ_{opt}	Optimal spread
δ_{res}	Client's reservation spread
Δ	Half the CBBT bid-ask spread
$f(\delta)$	Hit probability: Probability that a dealer wins an RfQ
γ	Risk-aversion parameter
IA	Information Asymmetry
MXF	Market external factors
n	Number of dealers in competition in the RfQ
P	Price quoted by the reference dealer
P_m	Market mid-price
P_{res}	Client's reservation price: Maximum (or minimum) price a client is willing to accept
PD	Price Discovery: RFQs issued without intent to trade
R	Revenue
R_0	Instantaneous flow value
R_T	End of day flow value
R_{rt}	Round-trip revenue
R_{t+h}	Short-term flow value
RF	RfQ Features: Side, volume, number of dealers, etc.
RfQ	Request for Quote
RS	RfQ Status: Hit, missed, passed, etc.
σ	Market volatility
s	RfQ side
v	RfQ volume
$\mathcal{Z}_t$	Information used by the dealer for estimating revenues at time t

Table 1: Notation
