# Supplementary material for: Causal interventions in bond multi-dealer-to-client platforms
Source: PLoS One. 2026 Jan 27;21(1):e0341369. doi: 10.1371/journal.pone.0341369 (PMC12844515; doi:10.1371/journal.pone.0341369)
Supplement: S3 Table — Feature importance across models for sell RfQs. (PDF) [file pone.0341369.s004.pdf]

# S3 Table. Feature importance

We examine the importance of variables across models. For the generative model, we rely on permutation importance (measured as the drop in AUC when permuting a given feature). For the logistic regression, we report standardized coefficients. For LightGBM, we use gain-based feature importance. Note that scales differ across methods (permutation $\Delta$AUC, standardized $\beta$, and tree gain are not directly comparable), so conclusions should be drawn from rankings rather than magnitudes.

| **Variable** | **Generative** | **Logistic R** | **LightGBM** |
|---|---|---|---|
| Spread | 0.001345 | 2.852968 | 1562002.945 |
| Volatility | 0.000190 | 0.009206 | 13619.651 |
| DV01 | 0.000044 | 0.878152 | 13810.043 |
| Frequency buy | 0.000024 | 0.001386 | 13918.092 |
| Frequency sell | 0.000036 | 0.042998 | 6969.025 |
| Average dealers | 0.000048 | 0.070142 | 22596.602 |
| Maturity | 0.000558 | 0.000269 | 22653.330 |
| Number of dealers | 0.000000 | 0.066780 | 88752.495 |
| DV01 exposure | 0.000000 | 0.212309 | 94853.899 |

Table 1: Feature importance across models for sell RfQs. Generative: permutation importance (drop in AUC). Logistic regression: standardized coefficients (absolute values). LightGBM: gain-based importance.
